# Relationship of oral bacterial number with medical hospitalization costs in analysis of Diagnosis Procedure Combination database from single institution in Japan

**DOI:** 10.1038/s41598-024-60733-z

**Published:** 2024-05-15

**Authors:** Hiromi Nishi, Mikihito Kajiya, Kouji Ohta, Hideo Shigeishi, Taiji Obayashi, Syuichi Munenaga, Nami Obayashi, Yukio Yoshioka, Masaru Konishi, Takako Naruse, Akihiro Matsumoto, Ayaka Odo, Masae Kitagawa, Toshinori Ando, Tomoaki Shintani, Tomoko Tokikazu, Natsumi Ino, Naoki Mihara, Naoya Kakimoto, Kazuhiro Tsuga, Kotaro Tanimoto, Hiroki Ohge, Hidemi Kurihara, Hiroyuki Kawaguchi

**Affiliations:** 1 Department of General Dentistry, Hiroshima University Hospital, 1-2-3 Kasumi, Minami-Ku, Hiroshima, 734-8553 Japan; 2 Department of Innovation and Precision Dentistry, Graduate School of Biomedical and Health Sciences, Hiroshima University, Hiroshima, Japan; 3 Department of Oral Laboratory Center, Hiroshima University Hospital, Hiroshima, Japan; 4 Department of Public Oral Health, Program of Oral Health Sciences, Hiroshima University, Hiroshima, Japan; 5 Department of Dental Hygiene, Ogaki Women’s College, Gifu, Japan; 6 Department of Oral Oncology, Graduate School of Biomedical Health Sciences, Hiroshima University, Hiroshima, Japan; 7 Department of Oral and Maxillofacial Radiology, Hiroshima University Hospital, Hiroshima, Japan; 8 Department of Oral and Maxillofacial Surgery, Graduate School of Biomedical and Health Sciences, Hiroshima University, Hiroshima, Japan; 9 Department of Medical Informatics, Graduate School of Biomedical and Health Sciences, Hiroshima University, Hiroshima, Japan; 10 Department of Orthodontics and Craniofacial Developmental Biology, Graduate School of Biomedical and Health Sciences, Hiroshima University, Hiroshima, Japan; 11 Department of Oral and Maxillofacial Pathobiology, Graduate School of Biomedical and Health Sciences, Hiroshima University, Hiroshima, Japan; 12 Department of Clinical Practice and Support, Hiroshima University Hospital, Hiroshima, Japan; 13 Department of Oral and Maxillofacial Radiology, Graduate School of Biomedical and Health Sciences, Hiroshima University, Hiroshima, Japan; 14 Department of Advanced Prosthodontics, Graduate School of Biomedical and Health Sciences, Hiroshima University, Hiroshima, Japan; 15 Department of Infectious Diseases, Hiroshima University Hospital, Hiroshima, Japan; 16 Dental Academy at Kudamatsu, Yamaguchi, Japan

**Keywords:** Hospitalization cost, Oral bacterial number, Oral conditions, Health care economics

## Abstract

Oral bacteria are known to be associated with perioperative complications during hospitalization. However, no presented reports have clarified the relationship of oral bacterial number with medical costs for inpatients. The Diagnosis Procedure Combination (DPC) database system used in Japan provides clinical information regarding acute hospital patients. The present study was conducted to determine the association of oral bacterial numbers in individual patients treated at a single institution with length of hospital stay and medical costs using DPC data. A total of 2369 patients referred by the medical department to the dental department at Hiroshima University Hospital were divided into the low (n = 2060) and high (n = 309) oral bacterial number groups. Length of hospital stay and medical costs were compared between the groups, as well as the associations of number of oral bacteria with Charlson comorbidity index (CCI)-related diseases in regard to mortality and disease severity. There was no significant difference in hospital stay length between the low (24.3 ± 24.2 days) and high (22.8 ± 20.1 days) oral bacterial number groups. On the other hand, the daily hospital medical cost in the high group was significantly greater (US$1456.2 ± 1505.7 vs. US$1185.7 ± 1128.6, P < 0.001). Additionally, there was no significant difference in CCI score between the groups, whereas the daily hospital medical costs for patients in the high group treated for cardiovascular disease or malignant tumors were greater than in the low number group (P < 0.05). Multivariate regression analysis was also performed, which showed that oral bacterial number, age, gender, BMI, cardiovascular disease, diabetes, malignant tumor, and hospital stay length were independently associated with daily hospitalization costs. Monitoring and oral care treatment to lower the number of oral bacteria in patients affected by cardiovascular disease or cancer may contribute to reduce hospitalization costs.

## Introduction

The main causes of prolonged hospital stay and high hospitalization costs for acute-care hospital patients are related to development of complications, such as an infectious disease^1^. It has also been shown that prolonged hospitalization of general internal medicine inpatients is associated with higher costs and complication rates because of nosocomial infections^2^, and that postoperative complications such as infection increased the cost of hospitalization for patients who underwent a simultaneous pancreas-kidney transplantation^3^. Additionally, a strong relationship among hospitalization costs, healthcare resource utilization, and postoperative complications following a lower anterior resection procedure for rectal cancer has been demonstrated^4^. Therefore, hospitalization cost and length of stay related to perioperative complications are important factors related to the quality of medical care provided by acute-care hospitals^5^.

Poor oral hygiene has been reported to be related to increased perioperative complications during hospitalization, while that noted in preoperative examinations was found to be independently associated with postoperative pneumonia following an esophageal cancer surgery procedure^6^. Furthermore, another study showed that poor oral health status related to missing teeth increased the risk of postoperative pneumonia after cardiovascular surgery as well as longer hospitalization^7^. On the other hand, improvement in oral hygiene has been found to have an association with decreased perioperative complications during hospitalization, leading to reduced hospitalization costs. Munro et al. reported that inpatients at Veterans Affairs hospitals in the United States who performed daily oral care by themselves twice daily showed a 92% reduction in hospital-acquired pneumonia, with a projected cost savings of US$2.84 million^8^. Oral bacterial number is known to be an indicator of the oral health environment of individuals and associated with onset of perioperative complications during hospitalization^9,10^, thus it may also be related to length of hospital stay and hospitalization expense in an acute-care setting.

The Diagnosis Procedure Combination (DPC) database system is a classification scheme developed in Japan for acute-care hospital inpatients^11,12^. DPC data provide inpatient clinical information, including unique identification data, age, gender, dates of admission and discharge, outcome status at discharge, major disease that consumed the most medical resources, comorbidity in association with International Classification of Diseases (ICD)-10 codes, and data linked with a lump-sum payment system^11^. Some investigators have used DPC data to analyze the relationships among main diseases and comorbidity, cost-effectiveness of procedures and medications, and factors affecting length of hospital stay for acute-care patients. However, even for institutions that have both medical and dental departments, data related to oral examinations and dental treatments performed at the dental department, as well as dental costs are not included in the DPC database for patients hospitalized by a medical department. As a result, no known studies have been conducted to clarify the association of oral environmental factors such as oral bacterial numbers in such hospitalized patients with medical costs related to quality of medical care using DPC data.

The present investigation was performed to determine whether oral bacterial numbers in patients hospitalized at a single acute-care institution had an association with length of hospital stay and/or medical costs. In addition, Charlson comorbidity index (CCI), proposed by Charlson et al., was used to predict the prognosis of each patient based on total score for the treated disease^13,14^, while the relationship of number of oral bacteria with CCI results was also evaluated in regard to mortality and disease severity. Finally, factors with possible influence on daily hospitalization costs were examined by use of multivariate regression analysis.

## Results

**Figure 1:**
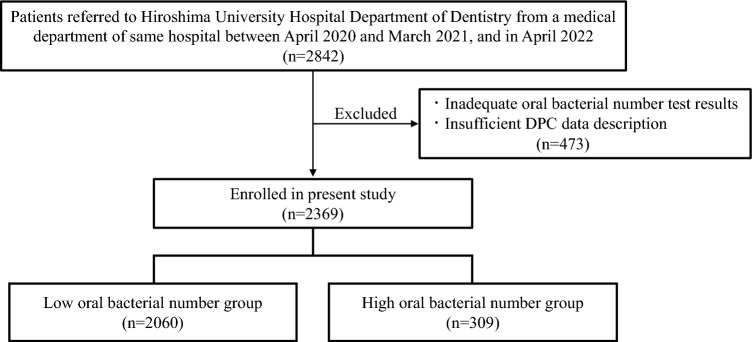
Flow of patient enrollment. Enrolled patients (n = 2369) were divided into the low (n = 2060) and high (n = 309) oral bacterial number groups.

A total of 2369 patients were enrolled, then divided into the low (n = 2060) and high (n = 309) oral bacterial number groups (Fig. 1), with their characteristics shown in Table 1. The number of patients between 18 and 64 years old was 842 (35.5%), and between 65 and 74 years old was 771 (32.5%), and 1503 (63.4%) were male, 1.7 times greater than female patients. As for BMI, the majority (n = 1448, 61.1%) were in a normal range (18.5–25), while 592 (25%) were classified as obese (> 25). The non-smoking group (no smoking history) with a smoking index of 0 consisted of 1498 (63.2%), while those with a smoking history (n = 412, 17.4%) had a smoking index of ≥ 600. Mean hospital stay was 24.1 ± 23.7 days and daily hospitalization cost was US$1222.9 ± 1192.4 (exchange rate ¥105 per US$1 in 2020). The mean oral bacterial number at admission was 4.83 × 10^6^ CFU/ml.

**Table 1:** Baseline clinical characteristics of enrolled patients (n = 2369).

Clinical parameters	
Age, years, n (%)	
18–64	842 (35.5)
65–74	771 (32.5)
75–84	572 (24.1)
> 85	185 (7.8)
Gender, male, n (%)	1503 (63.4)
BMI, n (%)	
< 18.5	330 (13.9)
18.5–25	1448 (61.1)
> 25	592 (25.0)
Brinkman index, n (%)	
Mild (0–199)	1498 (63.2)
Moderate (200–599)	262 (11.1)
Heavy (> 600)	412 (17.4)
Oral bacteria number (× 10^4^ CFU/ml)	483.0 ± 661.4
Oral bacteria number, n (%)	
Low (< 107 CFU/ml)	2060 (87.0)
High (≥ 107 CFU/ml)	309 (13.0)
Length of hospital stay, days	24.1 ± 23.7
Hospitalization costs per day, US$	1222.9 ± 1192.4

Characteristics of the patients classified based on severity of the 18 diseases used by the CCI from the obtained DPC data^13,14^ are shown in Table 2. Among the present cohort, 1138 (48.0%) had malignant tumors (localized and metastatic solid tumor cases), while another 574 (24.2%) had diabetes mellitus (DM).

**Table 2:** CMI scores and numbers of patients with specific diseases (enrolled n = 2369).

	CCI score	No	%
Diagnosis			
Myocardial infarction	1	42	1.8
Congestive heart failure		173	7.3
Peripheral vascular disease		72	3.0
Cerebrovascular disease		109	4.6
Dementia		28	1.2
Chronic pulmonary disease		13	0.6
Rheumatic disease		54	2.3
Peptic ulcer disease		79	3.3
Mild liver disease		229	9.7
DM without chronic complications		519	21.9
DM with chronic complications	2	55	2.3
Hemiplegia or paraplegia		9	0.4
Renal disease		24	1.0
Localized solid tumor*		950	40.1
Leukemia and lymphoma		229	9.7
Moderate or severe liver disease	3	11	0.5
Metastatic solid tumor	6	188	7.9
Acquired immunodeficiency syndrome		1	0.0
Age (years)			
≤ 40	0	148	6.2
41–50	1	197	8.3
51–60	2	311	13.1
61–70	3	613	25.9
71–80	4	739	31.2
> 80	5	362	15.3

*Patients with malignant neoplasm of the skin excluded. CCI: Charlson comorbidity index, DM: diabetes mellitus.

A comparison of clinical characteristics between the low (n = 2060) and high (n = 309) oral bacterial number groups is presented in Table 3. There were no significant differences regarding age, gender, or BMI between the groups. The length of hospitalization was 24.3 ± 24.2 days in the low and 22.8 ± 20.1 days in the high number group, which was not a significant difference. Total score for CCI was also not significantly different. On the other hand, daily medical cost was significantly higher in the high (US$1456.2 ± 1505.7) as compared to the low (US$1185.7 ± 1128.6) (P < 0.001) oral bacterial number group.

**Table 3:** Comparisons of various factors between low and high oral bacteria number groups.

Parameter	Low group (n = 2060)	High group (n = 309)	P value
Age, years, n (%)			0.10
18–64	736 (35.7)	105 (34.0)	
65–74	678 (32.9)	93 (30.1)	
75–84	479 (23.3)	93 (30.1)	
> 85	167 (8.1)	18 (5.8)	
Gender, male, n (%)	1307 (63.5)	196 (63.4)	0.99
BMI, n (%)			0.57
< 18.5	275 (13.4)	50 (16.2)	
18.5–25	1243 (60.3)	183 (59.2)	
> 25	511 (24.8)	71 (23.0)	
Brinkman index, n (%)			0.37
Mild (0–199)	1332 (64.7)	187 (60.5)	
Moderate (200–599)	200 (9.7)	42 (13.6)	
Heavy (> 600)	425 (20.6)	65 (21.0)	
Charlson Risk Index			0.44
Low (0)	30 (1.5)	4 (1.3)	
Medium (1–2)	241 (11.7)	34 (11.0)	
High (3–4)	531 (25.8)	68 (22.0)	
Very high (> 5)	1258 (61.1)	203 (65.7)	
Length of hospital stay (days)	24.3 ± 24.2	22.8 ± 20.1	0.31
Hospitalization cost per day, US$	1185.7 ± 1128.6	1456.2 ± 1505.7	< 0.001*

*P < 0.05 (statistically significant in t-test or chi-square test results).

**Figure 2:**
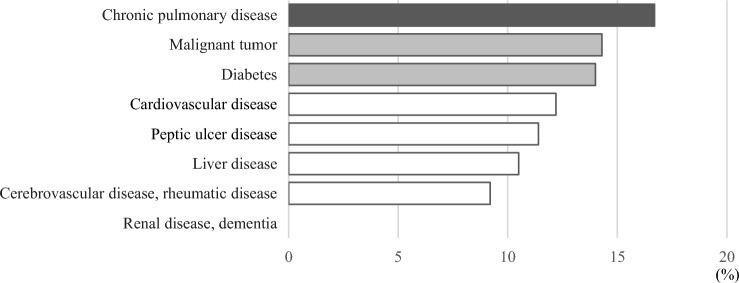
Percentages of patients with CCI-related conditions and high oral bacterial number. The percentage of patients with chronic pulmonary disease, a malignant tumor, or DM showed a greater tendency to have a high oral bacterial number as compared to those with the other conditions noted in the CCI.

**Figure 3:**
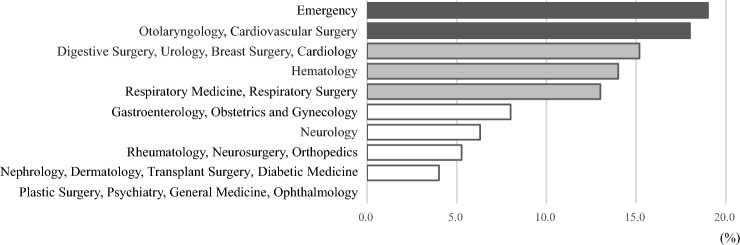
Percentages of patients treated at medical departments of Hiroshima University Hospital with high oral bacterial number. The percentage of patients with a high oral bacterial number and treated at the emergency department was highest among all departments.

The percentage of patients with a high oral bacterial number affected by chronic pulmonary disease, a malignant tumor, or DM tended to be higher as compared to those with the other conditions noted in the CCI (Fig. 2). Furthermore, the percentage of patients with a high oral bacterial number who received treatment at the emergency department was highest among all departments (Fig. 3). Table 4 shows length of hospital stay and daily hospital medical costs for patients with various conditions noted in the CCI, who were divided into the low and high oral bacterial number groups. There was no significant difference for number of hospital days between the two groups, whereas the daily medical cost for those with a high number of oral bacteria and affected by cardiovascular disease or a malignant tumor was greater as compared to patients with a low number (P < 0.05). Finally, multivariate regression analysis results showed factors that had an influence on daily hospitalization costs, which are presented in Table 5. Oral bacterial number was found to be independently associated with daily hospitalization costs (P = 0.011), while age, gender, BMI, cardiovascular disease, diabetes, malignant tumor, and hospital stay length were also found to be independent factors with influence on daily hospitalization costs.

**Table 4:** Comparison of CMI-related diseases between low and high oral bacterial number groups.

Parameter*	Low (n = 2060)	High (n = 309)	P value
Length of hospital stay (days)			
Cardiovascular disease	24.8 ± 21.1	17.4 ± 10.9	0.05
Cerebrovascular disease	22.1 ± 13.9	15.5 ± 14.2	0.15
Chronic pulmonary disease	20.6 ± 8.6	22 ± 12.5	0.83
Rheumatic disease	26.3 ± 16.3	39.2 ± 29.5	0.15
Peptic ulcer disease	23.7 ± 16.1	30.9 ± 39.7	0.15
Liver disease	27.4 ± 37.2	25.7 ± 18.9	0.82
Diabetes	25.7 ± 27.9	24.3 ± 18.1	0.67
Malignant tumor	25.1 ± 24.8	24.3 ± 20.1	0.70
Hospitalization cost per day, US$			
Cardiovascular disease	1423.8 ± 2100.0	3450.5 ± 4415.2	0.012*
Cerebrovascular disease	2457.1 ± 2252.4	2681.0 ± 2041.0	0.59
Chronic pulmonary disease	1509.5 ± 1249.5	3141.0 ± 3501.0	0.21
Rheumatic disease	1088.6 ± 906.7	720.0 ± 278.1	0.37
Peptic ulcer disease	855.2 ± 595.2	1399.0 ± 1621.0	0.05
Liver disease	986.7 ± 612.4	1160.0 ± 1745.7	0.31
Diabetes	1231.4 ± 1166.7	1299.0 ± 950.5	0.64
Malignant tumor	914.3 ± 604.8	1040.0 ± 747.6	0.011*

Patients with renal disease, dementia, or HIV were excluded, because none were classified into the high group.

*P < 0.05 (statistically significant in t-test results). CMI: Charlson comorbidity index.

**Table 5:** Multivariate regression analysis of factors with influence on daily hospitalization costs.

Parameter	Std error	t value	P value	95% confidence interval	
Age (years)	16.84	2.07	0.039*	1.75	67.83
Gender, male	262.81	− 2.38	0.020*	− 1140.75	− 109.75
BMI	58.15	4.71	< 0.0001*	159.98	388.10
Brinkman index	0.57	0.30	0.77	− 0.94	1.28
Oral bacterial number (× 104 CFU/ml)	10.34	2.53	0.011*	10.19	11.52
Diagnosis					
Dementia	1171.54	− 1.10	0.27	− 3585.47	1010.52
Cardiovascular disease	450.38	16.49	< 0.0001*	6541.55	8308.42
Cerebrovascular disease	661.69	0.45	0.65	− 999.04	1596.79
Chronic pulmonary disease	1657.41	− 0.61	0.54	− 4269.53	2232.52
Rheumatic disease	729.63	2.86	0.004*	658.11	3520.46
Peptic ulcer disease	660.24	1.45	0.15	− 340.77	2249.37
Liver disease	377.87	0.27	0.79	− 638.73	843.66
Renal disease	1546.78	− 0.18	0.86	− 3305.12	2762.91
Diabetes	281.21	2.03	0.043*	18.36	1121.56
Malignant tumor	258.74	4.41	< 0.0001*	633.19	1648.21
Length of hospital stay (days)	9.34	− 6.45	< 0.0001*	− 78.56	− 41.93

*P < 0.05 (statistically significant in regression analysis).

## Discussion

The DPC provides nationwide administrative hospitalization data obtained from Japanese hospital institutions, which have been used for various large-scale clinical studies^15–17^. Basic and clinical information for patients are included, such as date of birth, gender, residential postal code, dates of admission and discharge, route of admission, discharge status, diagnosis, surgical procedures, and various clinical scores. Previous reports have demonstrated a high validity of diagnoses and procedures based on inpatient records identified by DPC data^11^. Additionally, total hospitalization cost can also be calculated and studies have been performed to analyze cost-effectiveness for inpatients using a DPC-based payment system. Nakayama et al. examined the cost-effectiveness of inpatient management for hospitalized patients with cardiovascular diseases using DPC data, and found that management performed by female cardiologists was more cost-effective than that by male cardiologists^18^. In another study, Nishimura et al. evaluated the cost-effectiveness of two different bladder tumor resection methods using DPC data, which showed better cost effectiveness for a photodynamic diagnosis-assisted transurethral resection procedure as compared to a white light-assisted transurethral resection procedure^19^. The DPC system was introduced for inpatient care provided by medical departments, though the data do not include information regarding cost or treatment performed at a dental department during hospitalization. The present study is the first known to examine the relationship of oral bacterial number with medical inpatient information identified from the DPC system and the results showed an association of high oral bacterial count at the initial examination with increased daily hospitalization costs. Integration of results related to the oral environment and oral hygiene status of patients into the DPC data may provide a more comprehensive understanding of the economic impact of oral health care.

Previous studies have noted relationships of various perioperative complications with hospitalization costs, a review of which found that a surgical site infection (SSI) results in twice the cost for hospitalization as compared to patients without an SSI^20^. Furthermore, hospital-acquired and ventilator-associated pneumonia cases that required intensive care have been found to have increased hospitalization time and costs^21,22^. The impact of development of DM and infectious complications in DM patients on hospitalization costs has also been frequently reported^23–25^. In other studies, oral bacterial infection and poor oral health were shown to be risk factors related to surgical site infection after various cancer and cardiovascular surgical procedures^9,26–29^, while a relationship between oral microbial colonization and ventilator-associated pneumonia in patients in intensive care units has also been reported^30,31^. In other reports, oral bacteria were shown to be associated with onset of aspiration pneumonia after cancer surgery^32–34^. Furthermore, periodontal diseases caused by periodontal pathogens have been found to increase the risk of development of DM, with concomitant DM and periodontal disease shown to have adverse effects on glycemic control and complications, such as cardiovascular disease and end-stage renal disease^35,36^. Multivariate regression analysis results obtained in the present study showed that oral bacterial number is an independent factor with influence on daily hospitalization costs. The association of high number of oral bacteria with increased cost may be related to the effect on onset of perioperative complications. However, there may be other unknown confounding factors that influence the relationship, thus further study will be necessary.

The CCI provides categorization of patient comorbidities based on the ICD diagnosis codes found in hospital-based administrative data, and has been widely used for predictions of long-term prognosis and survival^37^. Recent reports indicate CCI as a valid indicator for predicting poor outcome in patients with interstitial lung disease, COVID19, or cardiac arrest^38–41^, while it has also been reported that CCI findings can be used to identify patients with potential to incur high hospitalization costs^42^. Additionally, CCI-related diseases can be identified from analysis of inpatient DPC data. Yamana et al. used a chart review to examine the level of agreement of DPC data-based diagnoses of diseases included in the CCI and the original diagnosis^43^. Although sensitivity was below 50% for some of the examined diseases, the specificity of diagnosis based on DPC data exceeded 96%. Therefore, to analyze the relationship of comorbidities with oral bacterial number, the CCI was used in the present study to perform analyses regarding diseases identified by DPC data. Although there was no significant difference regarding CCI score level between the high and low bacterial number groups, daily hospital medical costs for patients with a high number of oral bacteria treated for cardiovascular disease or a malignant tumor were greater as compared to patients treated for those conditions who showed a low number. Reducing the number of oral bacteria in patients affected by those conditions may result in decreased hospitalization costs.

Dental plaque removal by daily toothbrushing and oral health awareness are important factors for maintaining good oral hygiene^44,45^. Oral care has also been shown to prevent the incidence of perioperative complications in patients undergoing various cancer treatments and surgery under general anesthesia^46,47^, and can reduce the risk of onset of ventilator-associated pneumonia in patients in an intensive care unit^48^. In patients with diseases where poor oral hygiene is prevalent, such as head and neck cancers, as well as cerebrovascular disease, where oral microbiota can significantly influence outcome, the importance of oral care has been particularly emphasized^49–51^, with those reports indicating that an effective oral care strategy for patients with these conditions is crucial. Additionally, a multicenter study found that perioperative oral care during cancer treatment reduces hospitalization costs^52^. However, despite use of appropriate oral care, postoperative pneumonia has been reported to occur in various types of cancer cases^6,53^. While the present patients received standard perioperative oral care, it is important to note that oral bacterial count was found to fluctuate during the perioperative period. This finding highlights the critical need for enhanced oral care strategies, particularly for patients with cardiovascular disease or malignancy, as the analysis revealed that such patients with a higher number of oral bacteria incurred greater daily hospitalization costs. Improvements in methods used for oral care and its frequency, including monitoring the number of oral bacteria in patients affected by cardiovascular disease or a malignant tumor, may lead to cost reductions.

This study has several limitations. First, it was not possible to analyze the onset of complications during hospitalization, because the DPC data alone were insufficient to identify those. A future study is needed to investigate the relationship between complications onset and number of present bacteria. Second, results of oral examinations related to dental caries experience or periodontal status were not analyzed in this study, as costs for dental treatment performed at a dental department are not included in the DPC database. Dental caries experience and periodontal status are known to be associated with onset of various perioperative complications^7,46,54,55^. A future survey of such oral examination factors may provide additional evidence showing a relationship between oral bacterial number and cost. Third, a previous in vivo study that used mice showed that bacterial count temporarily decreases after eating^56^. Although, sample collection for bacterial count determination was avoided within one hour after eating in the present study, it will be important to collect samples from patients at the same time following a meal in order to examine diurnal variations of oral bacterial counts. Fourth, this was a cross-sectional study, which may have introduced bias. Finally, this study was conducted with patients treated at a single institution, which limits generalization of the findings. Epidemiological analysis to determine the relationship of improvement in oral bacteria number with hospitalization costs will be needed to generalize the present findings.

In summary, this is the first study to reveal that daily medical costs for patients with a high number of oral bacteria were greater as compared to those with a low number. Notably, patients with a high oral bacterial number, and hospitalized for a malignant tumor or cardiovascular disease were found to incur greater daily medical costs as compared to those with a low number. Monitoring and oral care treatment to improve oral bacterial number in patients affected by cardiovascular disease or cancer may contribute to reduce costs related to hospitalization.

## Methods

### Study variables and data acquisition

The DPC database system was introduced for acute-care hospitals in Japan in 2003^57^. Data included in this system include patient demographics and selected clinical information, such as admission and discharge status, diagnosis, surgeries and procedures performed, medications, payments for specific conditions, and hospitalization costs^58^. For the present study, age, gender, body mass index (BMI), smoking coefficient, length of hospital stay, hospitalization costs, and comorbidities were collected as clinical information. Daily medical costs were calculated as the sum of the bundled medical payment without any food fee, then divided by duration of hospital stay. Hospitals keep DPC data separate from dental department information for the same patients. Inpatients referred from a medical department for a dentistry examination are treated as outpatients in the dental department, and the DPC database does not include oral examination, dental treatment, or dental cost information related to oral care performed by the dental department during hospitalization. The Brinkman Index was used for smoking coefficient to predict the effects of smoking on the human body and calculated by multiplying the average number of cigarettes smoked per day by number of years smoked^59^. The oral bacterial number used was determined at the first visit to the dental department.

### Patients

Hospitalized patients 18 years or older who were referred from a medical department of Hiroshima University Hospital to the dental department of the same hospital in the period from April 2020 to April 2022 were initially considered for participation in this retrospective study. However, it was not possible to regularly determine oral bacterial numbers with a bacterial counter for patients from April 2021 to March 2022, thus there was a lack of data regarding those numbers in that period. Therefore, a total of 2842 hospitalized patients of that age who were referred from a medical department of Hiroshima University Hospital to the dental department of the same hospital between April 2020 and March 2021, and also in April 2022 were enrolled as subjects in this retrospective study. Four hundred seventy-three with incomplete DPC data or errors in oral bacteriology findings noted during hospitalization were excluded (Fig. 1). Patients hospitalized more than once during the survey period were separately analyzed for each hospitalization. The opportunity for questions and comments was ensured through an opt-out approach, and approved by the Ethics Committee of Hiroshima University Hospital for the present study (approval number E-epidemic 2086). The Ethics Committee of Hiroshima University Hospital waived the need for informed consent. All data analysis results were blinded and accessed for research purposes from November 1, 2022 to December 20, 2022.

### Oral bacterial counts

Oral bacterial numbers were determined during the initial examination at the dental department, with sample collection within one hour after eating avoided because of possible postprandial effects. Bacteria were obtained by rubbing the tongue surface six times with a sterile cotton swab, according to the manufacturer's instructions^60^. Microorganisms in the obtained specimens were determined using a device that provides quantification based on electrical impedance (Panasonic Healthcare Holdings Co., Ltd., Tokyo), with oral bacterial numbers automatically determined. For the present study, an oral bacterial count greater than 10^7^ CFU/mL was considered a high level, according to findings of a previous study that used the same instruments^61,62^. Based on number of oral bacteria present, the patients were classified into the low (< 10^7^ CFU/ml, n = 2060) and high (≥ 10^7^ CFU/ml, n = 309) groups (Fig. 1).

### Severity assessment

Patient diagnoses and comorbidities were classified according to the criteria of the CCI using DPC data^13,14^. The CCI is widely employed for epidemiological and cohort studies to process large amounts of data^63^. For that, multiple comorbidities are scored individually and the total score is used to evaluate severity. The following ten conditions each receive one point: (1) definite or probable history of myocardial infarction (electrocardiogram and/or enzyme changes); (2) congestive heart failure, shown by exertional or paroxysmal nocturnal dyspnea, and patient has responded to digitalis, diuretics, or afterload reducing agents; (3) peripheral vascular disease, shown by intermittent claudication or previous bypass performed for chronic arterial insufficiency, history of gangrene or acute arterial insufficiency, or untreated thoracic or abdominal aneurysm (≥ 6 cm); (4) history of cerebrovascular accident with minor or no residual or transient ischemic attacks; (5) dementia as a chronic cognitive deficit; (6) chronic obstructive pulmonary disease; (7) connective tissue disease; (8) peptic ulcer disease, shown by any history of treatment for ulcer disease or history of ulcer-related bleeding; (9) mild liver disease shown by chronic hepatitis or cirrhosis without portal hypertension; and (10) uncomplicated DM. The following six conditions each receive two points: (1) hemiplegia; (2) renal disease, shown by the patient on dialysis, or with post-kidney transplant status, uremia, or creatinine > 3 mg/dL (0.27 mmol/L); (3) end-organ damage DM; (4) localized solid tumor; (5) leukemia; and (6) lymphoma. Severe liver disease shown by cirrhosis and portal hypertension with variceal bleeding history, cirrhosis, and portal hypertension but no variceal bleeding history receives three points. The following conditions receive six points: (1) metastatic solid tumor and (2) AIDS. The CCI also includes age as an adjustment factor for prognosis, with one point added for every ten years after 41 years and five points added for patients over 80 years. Using the CCI, patients with a total score of 1–2 points were placed in the Medium group, with 3–4 points in the High group, and 5 or more points in the Very High group^13,64^.

### Statistical analysis

Values are expressed as standard deviation (SD), mean, or median for continuous variables, and frequency and percentage for discrete variables (SAS Institute Inc., Cary, NC, USA). The statistical significance of between-group differences was determined using an unpaired t-test or Mann–Whitney's U test for continuous variables, or Fisher’s exact test, or if necessary, a χ^2^ test for discrete variables. The enrolled patients were classified into the high and standard groups based on number of oral bacteria (regardless of smoking history), then univariate analysis was used to evaluate DPC data for gender, age, hospitalization days, preoperative hospitalization days, postoperative hospitalization days, length of stay, number of complications, and hospitalization medical costs for each group. Specific multivariate regression analysis was conducted to identify factors with influence on daily hospitalization costs. This analysis used cost of daily hospitalization as the dependent variable and also included age, gender, BMI, Brinkman Index, oral bacterial count, length of hospital stay, and comorbidities (dementia, cardiovascular disease, cerebrovascular disease, chronic pulmonary disease, rheumatic disease, peptic ulcer disease, liver disease, renal disease, diabetes, malignant tumor) as independent variables. Furthermore, variance inflation factors (VIFs) were calculated to assess multicollinearity and explanatory variables with a VIF < 2, and were included in the analysis, with multicollinearity also used to ensure the independence of the variables. A P value < 0.05 was considered to indicate statistical significance.

### Ethics declaration

All procedures involving human participants were performed in accordance with relevant guidelines and regulations, as well as the ethical standards of the Ethics Committee of Hiroshima University Hospital, and done in accordance with the 1964 Helsinki Declaration and its later amendments or comparable ethical standards. This was a retrospective study, thus the need for written informed consent was formally waived.

## Data Availability

The data sets generated during and/or analyzed for the present study are available from the corresponding author upon reasonable request.
